# Competition with insectivorous ants as a contributor to low songbird diversity at low elevations in the eastern Himalaya

**DOI:** 10.1002/ece3.6196

**Published:** 2020-03-30

**Authors:** K. Supriya, Trevor D. Price, Corrie S. Moreau

**Affiliations:** ^1^ School of Life Sciences Arizona State University Tempe AZ USA; ^2^ Committee on Evolutionary Biology University of Chicago Chicago IL USA; ^3^ Department of Ecology and Evolution University of Chicago Chicago IL USA; ^4^ Departments of Entomology and Ecology & Evolutionary Biology Cornell University Ithaca NY USA

**Keywords:** ant exclusion, inter‐clade competition, macroecology, molecular diet analyses

## Abstract

Competitive interactions between distantly related clades could cause complementary diversity patterns of these clades over large spatial scales. One such example might be ants and birds in the eastern Himalaya; ants are very common at low elevations but almost absent at mid‐elevations where the abundance of other arthropods and insectivorous bird diversity peaks. Here, we ask if ants at low elevations could compete with birds for arthropod prey. Specifically, we studied the impact of the Asian weaver ant (*Oecophylla smaragdina*), a common aggressive ant at low elevations. Diet analysis using molecular methods demonstrate extensive diet overlap between weaver ants and songbirds at both low and mid‐elevations. Trees without weaver ants have greater non‐ant arthropod abundance and leaf damage. Experimental removal of weaver ants results in an increase in the abundance of non‐ant arthropods. Notably, numbers of Coleoptera and Lepidoptera were most affected by removal experiments and were prominent components of both bird and weaver ant diets. Our results suggest that songbirds and weaver ants might potentially compete with each other for arthropod prey at low elevations, thereby contributing to lower insectivorous bird diversity at low elevations in eastern Himalaya. Competition with ants may shape vertebrate diversity patterns across broad biodiversity gradients.

## Graphical abstract

Competitive interactions between distantly related clades could cause complementary diversity patterns of these clades over large spatial scales. One such example might be ants and birds in the eastern Himalaya; ants are very common at low elevations but almost absent at mid‐elevations where the abundance of other arthropods and insectivorous bird diversity peaks. Here, we present some evidence for dietary overlap between Asian Weaver ants and birds at low elevations and show that removal of weaver ants results in increase in non‐ant arthropod population on trees.

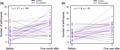

## 1 INTRODUCTION

Biotic interactions such as mutualism, commensalism, competition, and predation affect diversity and distribution of species at different scales in different ways (Araújo & Rozenfeld, 2014; Lany et al., 2018; Nelsen, Ree, & Moreau, 2018; Schemske, Mittelbach, Cornell, Sobel, & Roy, 2009; Wisz et al., 2013). While the role of these interactions in shaping large‐scale biodiversity patterns is often acknowledged, it is rarely tested (McCain & Grytnes, 2010; Schemske et al., 2009; Wisz et al., 2013). This paucity of studies may be attributed to the fact that biotic interactions are often difficult to quantify (McCain & Grytnes, 2010) and that cause and effect can be difficult to distinguish in the relationship between biotic interactions and species diversity (Fischer, 1960; Schemske et al., 2009). However, there is a growing need to study the role of these interactions in shaping diversity patterns in the light of predicted shifts in species ranges due to climate change (Anderson, 2017; Blois, Zarnetske, Fitzpatrick, & Finnegan, 2013; Gavish et al., 2017; Valiente‐Banuet et al., 2015; Wisz et al., 2013), especially as the strength and effects of these interactions are themselves susceptible to climate change (Faldyn, Hunter, & Elderd, 2018; Tylianakis, Didham, Bascompte, & Wardle, 2008).

One of the key biotic interactions that can influence diversity patterns in various ways is competition. Competition may reduce diversity by competitive exclusion (Goldberg & Barton, 1992; Valone & Brown, 1995) or enhance diversity through increasing specialization of species (Abbott, Abbott, & Grant, 1977; Emerson & Kolm, 2005; Futuyma & Moreno, 1988). Closely related taxa often compete for similar resources, and these competitive interactions can influence range limits of these taxa (Jankowski, Robinson, & Levey, 2010; Pasch, Bolker, & Phelps, 2013; Terborgh & Weske, 1975). However, interactions between distantly related taxa also have major impacts on diversity patterns. Although a few studies have demonstrated competition between distantly related taxa (Brown & Davidson, 1977; Eriksson, 1979; Hochberg & Lawton, 1990; Jennings, Krupa, Raffel, & Rohr, 2010; Palmeirim, Gorchoy, & Stoleson, 1989) and the effect of competition between distantly related taxa on diversity patterns has been inferred from the fossil record (Jablonski, 2008), this subject has not received much attention in macroecology. In fact, the presence of closely related species in a community has been used as evidence against the role of competition, emphasizing instead abiotic filtering (Gómez, Bravo, Brumfield, Tello, & Daniel, 2010; Tucker et al., 2017; Webb, Ackerly, Mcpeek, & Donoghue, 2002). However, recent advances in ecological coexistence theory imply that strong differences in competitive ability between distantly related competitors could cause competitive exclusion even if niches are substantially different (Chesson, 2000; Gerhold et al., 2015; Mayfield & Levine, 2010). Indeed, many empirical studies support this model for ecological coexistence (Germain, Weir, & Benjamin, 2016; Venail et al., 2014).

Here, we present observational and experimental evidence that suggests a role for ecological competition between two distantly related clades in shaping their complementary diversity patterns: songbirds (Phylum Chordata, Class Aves, Subfamily Oscines) and ants (Phylum Arthropoda, Class Insecta, Family Formicidae). While several studies have presented evidence for competition or amensalism between ants and birds (Table S1), they have not assessed the effect of these interactions on patterns of species diversity. Ants are important predators of other arthropods, especially in tropical and subtropical lowland forests (Floren, Biun, & Linsenmair, 2002; Sam, Koane, & Novotny, 2014). They have been experimentally shown to reduce numbers of other arthropods (Karban, Grof‐Tisza, & Holyoak, 2017; Piñol et al., 2010). However, ants are absent or are very low in abundance in tropical and subtropical montane cloud forests across the world, for reasons that remain unclear (Janzen, 1973; Longino, Branstetter, & Colwell, 2014; Samson, Rickart, & Gonzales, 1997). The elevation at which cloud forests are found varies with latitude and other geographic factors, but the lower elevational limit usually lies between 1,500 and 2,500 m and upper limit ranges from 2,400 to 3,300 m (Stadtmüller, 1987). One possible explanation for absence of ants from cloud forests is that ground‐nesting ants cannot persist in places that are wet throughout the year, whereas arboreal nesting ants cannot survive freezing temperatures (Janzen, 1973; Samson et al., 1997; Wheeler, 1917). On the other hand, although capable of living at low and higher elevations, carabid beetles (Maveety, Browne, & Erwin, 2013; Wilson, 1987), songbirds (Price et al., 2014), and small mammals (Heaney, 2001) are often very diverse and abundant in these cloud forests. One mechanism for this diversity and abundance maxima in cloud forests could be the lack of competition with ants for arthropod prey (Heaney, 2001; Price et al., 2014). We investigated this hypothesis in the eastern Himalaya.

**FIGURE 1:**
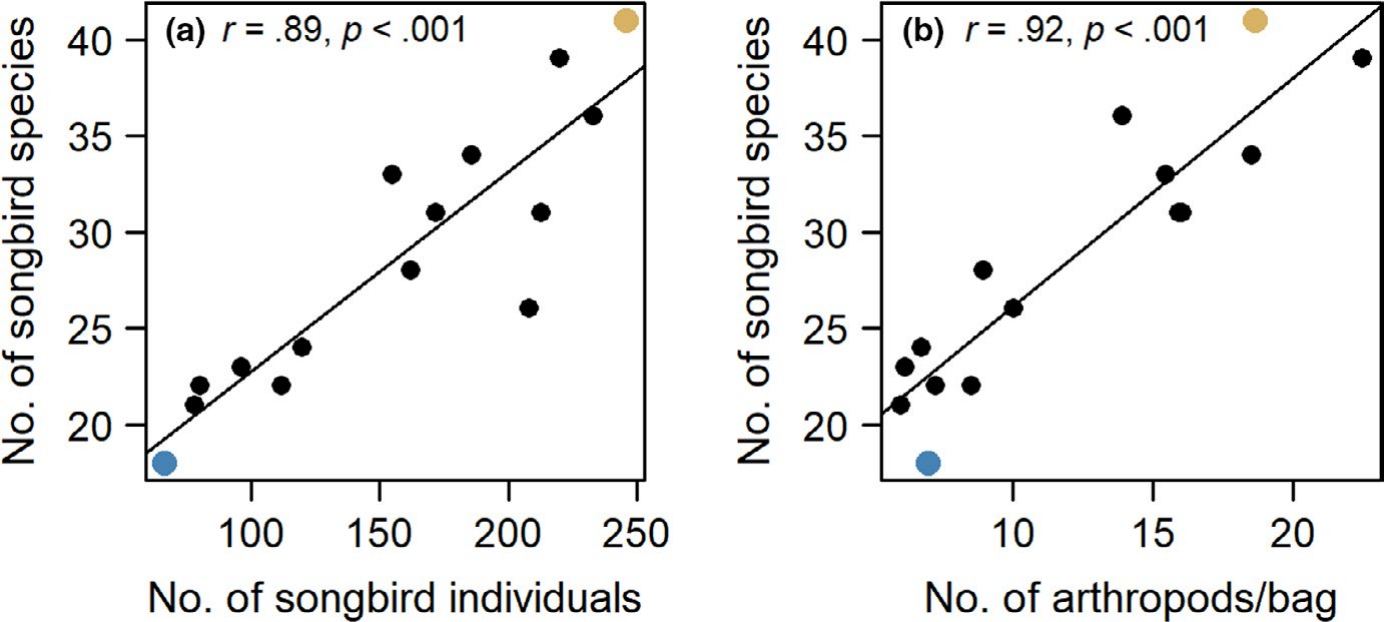
(a) Correlation between number of songbird species and individuals along an elevational gradient in the eastern Himalaya. (b) Correlation between number of songbird species and arthropod abundance along the same gradient. Site at 200 and 2,000 m elevation examined further in this study shown in blue and yellow, respectively. Based on data in Price et al. (2014)

In the eastern Himalaya, breeding songbird diversity peaks in the cloud forests at elevations between 1,200 and 2,000 m. Various historical and dispersal hypotheses, including greater area, dispersal from both above and below, and greater time for diversification at mid‐elevations (associated with climatic niche conservation) have little support as an explanation for the mid‐elevation peak in bird diversity (Price et al., 2014). The peak consists largely of small insectivorous bird species and is associated with greater resource (i.e., arthropod) abundance, potentially supporting more individuals, and hence, more species (Price et al., 2014; Schumm, White, Supriya, & Price, 2019; Figure 1; Figures S1 and S2). By contrast, ants are almost absent in the cloud forests at mid‐elevations, even though they are highly abundant and diverse at the low elevations (Figure 1; Figures S1 and S2; Ghosh‐Harihar, 2013; Price et al., 2014). Among ants, low elevations are dominated by an arboreal insectivorous species, the Asian weaver ant *Oecophylla smaragdina* which disappears at about 900 m elevation (K. Supriya pers. obs.). Weaver ants forage both on the trees and on the ground, move between trees through canopy connections, and are highly aggressive (Basu, 1997; Peng & Christian, 2005; Van Mele, 2008). We evaluated the possibility that the dominance of weaver ants at low elevations contributes to the lower diversity of birds at these elevations due to competition for food resources.

To test whether weaver ants and birds might compete for resources at the low elevation, we first assessed if there is dietary overlap between weaver ants and birds. A necessary precondition for competition between two taxa is significant overlap in the use of the same limiting resource (Brown & Davidson, 1977). Previous research suggests that arthropods are a limiting resource for songbirds in the eastern Himalaya (Price et al., 2014; also see Figures S1 and S2). Here, we compared the diet of weaver ants and birds at the low elevation where they co‐occur, and at higher elevations where weaver ants are absent, to assess dietary overlap. Next, we compared arthropod abundance and leaf damage due to insect herbivory on trees with and without weaver ants and conducted a weaver ant removal and exclusion experiment using a paired design to assess whether weaver ants significantly reduce arthropod abundance on trees.

## 2 METHODS

### 2.1 Study sites

All fieldwork was conducted over April–June of 2014–2016. Ants and birds were studied at the moist subtropical broadleaved forests at low elevations in Chapramari Wildlife Sanctuary (26.8858°N, 88.8341°E, ~200 m) in West Bengal, India. Bird diets were studied at Chapramari and in the temperate broadleaved cloud forests at the mid‐elevation site of Neora Valley National Park (27.0611°N, 88.7707°E, ~2,000 m) in West Bengal. Bird diets were also studied at 1,200 m elevation in Neora Valley National Park (27.03°N, 88.78°E) and at 2,300 m (27.42°N, 88.20°E), 2,700 m (27.43°N, 88.19°E), 3,200 m (27.45°N, 88.17°E) elevations in Khangchendzonga National Park in Sikkim, India.

### 2.2 Molecular diet analysis

#### 2.2.1 Field and laboratory methods

To examine bird diets, between April‐June 2016 we caught 103 birds and successfully collected 41 fecal samples at 200 and 2,000 m elevations at the Bengal sites (Table S2). We caught an additional 107 birds and collected 82 fecal samples at 1,200, 2,300, 2,700, and 3,200 m elevations in May 2017. All fecal samples were stored in 95% ethanol until DNA extraction. We used Qiagen QIAamp DNA Stool Mini kit to extract DNA with some minor modifications in the protocol, following Zeale, Butlin, Barker, Lees, and Jones (2011).

To examine weaver ant diet, we collected items of food that the weaver ants were carrying to their nest between April‐June 2016. We collected food items from 25 different colonies, either for an hour or until we had collected 10 items, whichever happened first. We extracted DNA from these samples by taking a small part of each of the prey items, crushing it and then using the Qiagen DNeasy blood and tissue kit and following the manufacturer's protocol for DNA extraction. We carried out a 25 μl PCR (polymerase chain reaction) to amplify a segment of 16S rDNA using the lns16S‐short primer pair (5′‐3′ TRRGACGAGAAGACCCTATA; ACGCTGTTATCCCTAAGGTA) described by Clarke, Soubrier, Weyrich, and Cooper (2014). We used this primer set because it is invertebrate‐specific, so successful DNA amplification indicates presence of prey DNA in the bird fecal DNA extract. We sent successful PCR products to the Sequencing core at the University of Illinois at Chicago, Chicago, Illinois, for barcoding, pooling, size selection, and sequencing on an Illumina Miniseq platform. All PCR products had strong primer dimer bands that were removed at the sequencing core before sequencing with a procedure that selected for fragments longer than 150 bp (see Supplementary Methods for further details).

#### 2.2.2 Data analysis

We followed the dada2 pipeline (Callahan et al., 2016) to construct an amplicon sequence variant (ASV) table and then created a FASTA file with all the unique sequences recovered from our samples. We used blastn in blastplus to BLAST this FASTA file against the NCBI nr database and used the NCBI‐taxcollector script (Dias et al., 2014) to get detailed taxonomic information for the top hit of our BLAST results. After filtering out bacteria and other contaminating sequences, we estimated overlap between the diets of weaver ants and birds at low and mid‐elevations at various taxonomic levels using EcoSimR (Gotelli, Hart, & Ellison, 2015) and visualized the overlap using venn diagrams and nonmetric multidimensional scaling (NMDS) in R (R Core Team, 2014). EcoSim R is used to estimate niche overlap between species and compare it to a null distribution of niche overlap given information on resource utilization (in columns) by each species (rows). The algorithm randomizes resource utilization for each species by reshuffling the row values and generates a null distribution of niche overlap. In our case, we considered birds at low elevations (*N* = 18), birds at middle elevations (*N* = 15), and weaver ants (*N* = 25) at low elevations as “species” and used the frequency of occurrence of different orders or families in diets as the “resources.” All the analyses were done in the R programming environment and in the shell using the R packages dada2 (Callahan et al., 2016), DECIPHER (Wright, 2016), VennDiagram (Chen & Boutros, 2011), ggplot2 (Wickham, 2011), vegan (Oksanen et al., 2018), and EcoSimR (Gotelli et al., 2015). All scripts and details of the steps are available as supplements to this paper.

### 2.3 Effect of weaver ants on arthropod abundance

#### 2.3.1 Field methods

In June 2014 and May 2015, we measured arthropod abundance at 17 pairs of trees with weaver ants present and trees without weaver ants, where the trees were paired by species, height, and girth. To measure arthropod abundance, we beat the foliage of a tree with a stick and collected all the insects that fell on an upturned umbrella (≈100 cm diameter), which is similar to the method used by Piñol, Espadaler, and Cañellas (2012). We beat the foliage three times before collecting arthropods in vials containing 95% ethanol and repeated the process at another part of the tree. Later in the field camp, we counted all the collected arthropods, measured body length to the nearest mm, and classified them to taxonomic order. To control for observer bias, the person counting the arthropods was unaware of the presence or absence of weaver ants on the source tree. We also estimated leaf damage on the pairs of trees as a longer‐term proxy of insect herbivore abundance, with greater arthropod abundance implied by higher leaf damage. We used two methods to assess leaf damage: (a) visual estimation of % absent on 10 leaves from different parts of each tree and (b) visual estimation of % absent on each leaf on a short (~0.5 m) clipped branch of the tree.

In April–June 2015, we carried out a weaver ant removal and exclusion experiment using 15 pairs of trees, paired by species, height, and girth. On trees in the experimental treatment group, we removed all weaver ant nests using a tree pruner and then applied a band of Tanglefoot™ around the trunk of the tree at about 1 m height from the base of the tree. We plugged all the holes in the trunk crevices under the Tanglefoot band with cotton. We were unable to completely remove or exclude weaver ants from 6 out of 15 treatment trees because of the nature of the bark of the tree trunk or the canopy of the tree. On trees receiving the control treatment, we attached a band of brown paper to make them appear similar to trees receiving the experimental treatment and pruned a few branches to mimic the disturbance caused by the removal of weaver ant nests. We measured arthropod abundance at each of these trees using beating and branch‐clipping (Ozanne, 2005) before and one month after the experimental treatments. We also recorded leaf damage as an index of insect herbivory on the clipped branch and on 16 random leaves at each tree. We recorded arthropod abundances 1 year later in April–June 2016, but by that time a number of trees had gained or lost weaver ants to an uncertain degree; results are presented in the supplement.

#### 2.3.2 Data analysis

We used paired *t* tests to compare arthropod abundance and leaf damage between trees with or without weaver ants. Arthropod abundance data were log‐transformed with one added to all values to avoid zeroes in the data. We also used paired *t* tests to compare the change in arthropod abundance and leaf damage over the period of one month and over the period of one year between trees in the weaver ant exclusion and control treatments. Since we were interested in the effect of weaver ants on other arthropods, we removed the number of all ants from our arthropod count. We also removed the number of insects belonging to the suborder Homoptera, because weaver ants form mutualistic associations with Homopterans (Crozier, Newey, Schlüns, & Robson, 2009; Peng & Christian, 2005). All analyses were done in the RStudio programming environment (R Core Team, 2014).

## 3 RESULTS

### 3.1 Molecular diet analysis

Of the 41 fecal samples collected at 200 and 2,000 m elevations, we were able to amplify and sequence prey DNA from 33 samples in total, from five bird species at low elevations and nine different bird species at mid‐elevations (Table S2) and all 25 weaver ant food samples. We were also able to amplify and sequence prey DNA from 36 additional bird fecal samples at higher elevations in Sikkim and 2 additional samples at 1,200 m elevation. Even though the primer set we used was supposed to be invertebrate‐specific, it amplified some vertebrate taxa such as Squamata as well (Table S3). We recovered 1,331 amplicon sequence variants (ASVs) from the dada2 pipeline. Of these, 1,072 sequences yielded BLAST matches which reduced to 980 sequences after filtering for bacteria and contaminants (162 from birds at the 200 m elevation, 224 from birds at the 2,000 m elevation, 325 from weaver ants, and 333 from birds at 1,200, 2,300, 2,700, and 3,200 m elevations; note that this is greater than 980 because of overlap in ASVs among these groups).

**FIGURE 2:**
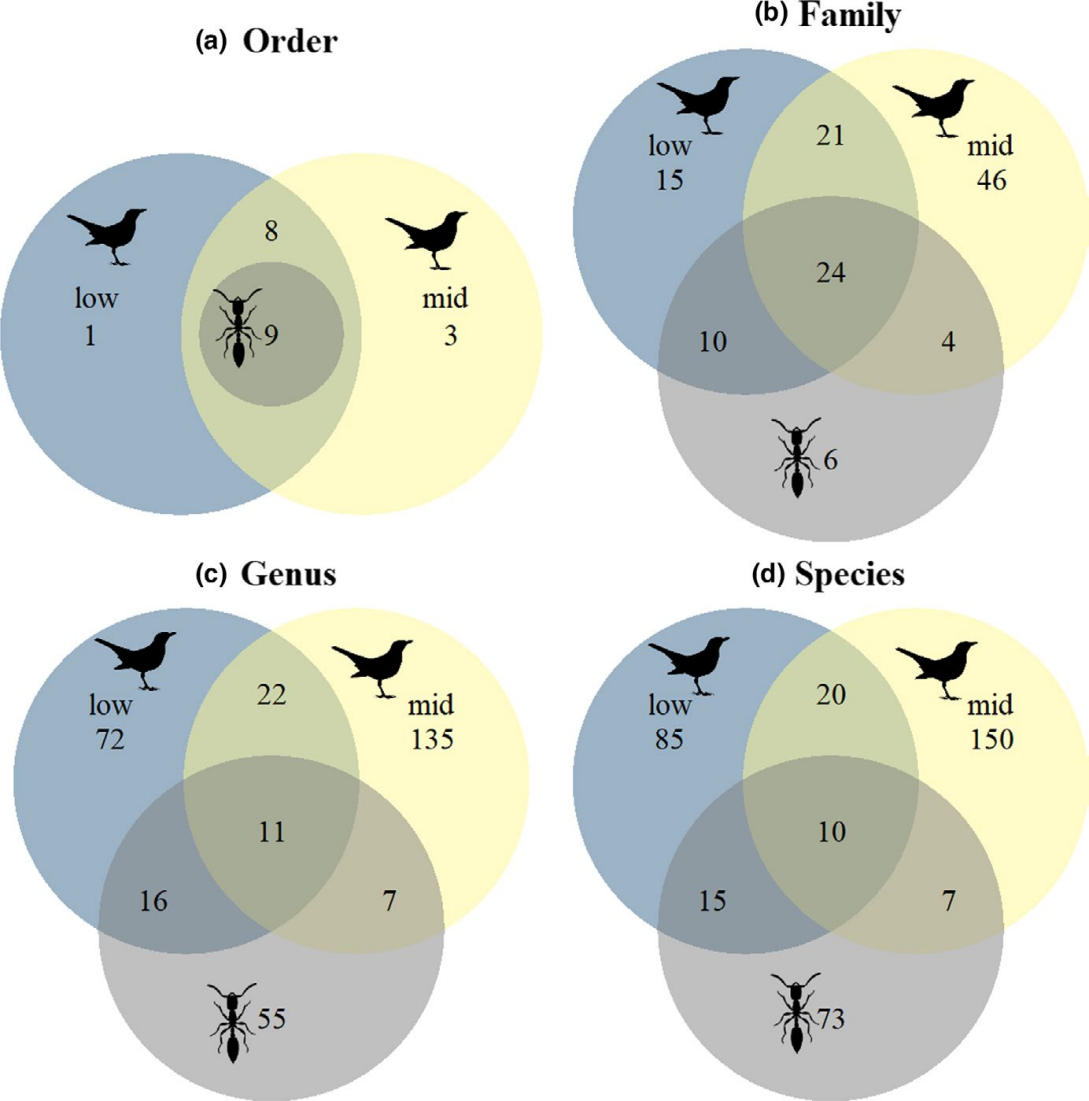
Venn diagram showing diet overlap at multiple taxonomic levels as assessed with molecular metabarcoding between weaver ants, birds at low elevations, and birds at mid‐elevations. Bird and ant silhouettes taken from http://phylopic.org. Anthony Caravaggi produced the bird silhouette

The most frequent orders in the bird diet at all elevations were Lepidoptera and Coleoptera (Figure S3; Table S3). At 200 m, Lepidoptera was detected in 94% and Coleoptera in 88% of the samples. Molecular diet analyses confirmed that Lepidoptera (69%) and Coleoptera (48%) are common in weaver ant diet, in addition to Blattodea, Hemiptera, and Hymenoptera (all 61%). Each of the orders present in weaver ant diet were also found in bird diets (Figure 2; Figure S4; Tables S3–S6) but birds had consumed animals in nine additional orders. These included larger animals such as centipedes (order Scolopendromorpha, 33%), annelids (order Haplotaxida, 11%), molluscs (class Gastropoda, 5.5%), and lizards (order Squamata, 5.5%) as well as some small arthropods such as springtails (order Entomobryomorpha, 11%), stoneflies (Plecoptera, 22%), booklice (Psocoptera, 5%), thrips (Thysanoptera, 5%), and earwigs (Dermaptera, 11%). At elevations between 2,000 and 3,200 m (where weaver ants are absent), we identified all but one of the 18 orders present at the low elevation, plus three more (lacewings [order Neuroptera, 44%], nemertean worms [order Monostilifera, 6%], and mayflies [order Ephemeroptera, 6%]).

Our EcoSimR results showed that the diets of birds at low elevations and weaver ants overlapped significantly more than expected from random resource utilization at all taxonomic levels, albeit not at the ASV level. Diet of birds from low and mid‐elevations overlapped significantly at order, family, and ASV levels. Diet of birds from mid‐elevations and the diet of low elevation weaver ants (weaver ants are absent at mid‐elevation) did not overlap significantly at finer taxonomic scales (Table S7). Lower diet overlap at finer taxonomic scales could be a function of sampling as indicated by the absence of asymptote in accumulation curves at finer taxonomic levels (Figure S5) or the difference in arthropod species across the elevational gradient.

### 3.2 Effect of weaver ants on arthropod abundance

**FIGURE 3:**
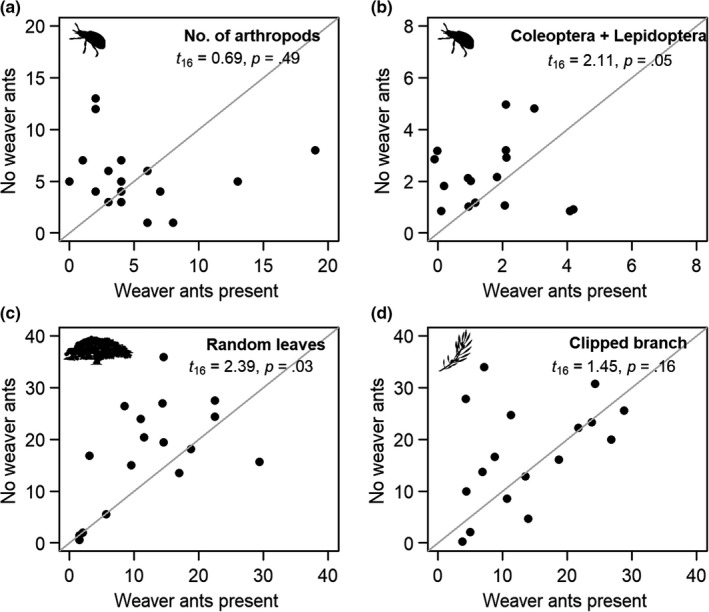
(a–d) Data from 34 trees paired for size and species, where one tree had weaver ants and the other did not. The gray line shows a slope of 1.0, that is, points on this line indicate pairs that do not differ. (a) Number of arthropods (excluding ants and suborder Homoptera). (b) Number of arthropods belonging to the orders Lepidoptera and Coleoptera. (c) Percent leaf damage estimated on 10 leaves distributed around the tree. (d) Percent leaf damage estimated on a clipped branch

We found no significant difference between number of arthropods on trees with or without weaver ants (Figure 3a). However, the number of insects belonging to orders Coleoptera and Lepidoptera, the two most common orders in bird diet at all elevations, was 1.7× higher on trees without weaver ants than on trees with weaver ants (Figure 3b). Leaf damage estimated from 10 leaves on each tree was significantly greater on trees without weaver ants than on trees with weaver ants (Figure 3c) and showed a nonsignificant trend in the same direction on the clipped branches from these trees (Figure 3d).

### 3.3 Weaver ant removal and exclusion experiment

**FIGURE 4:**
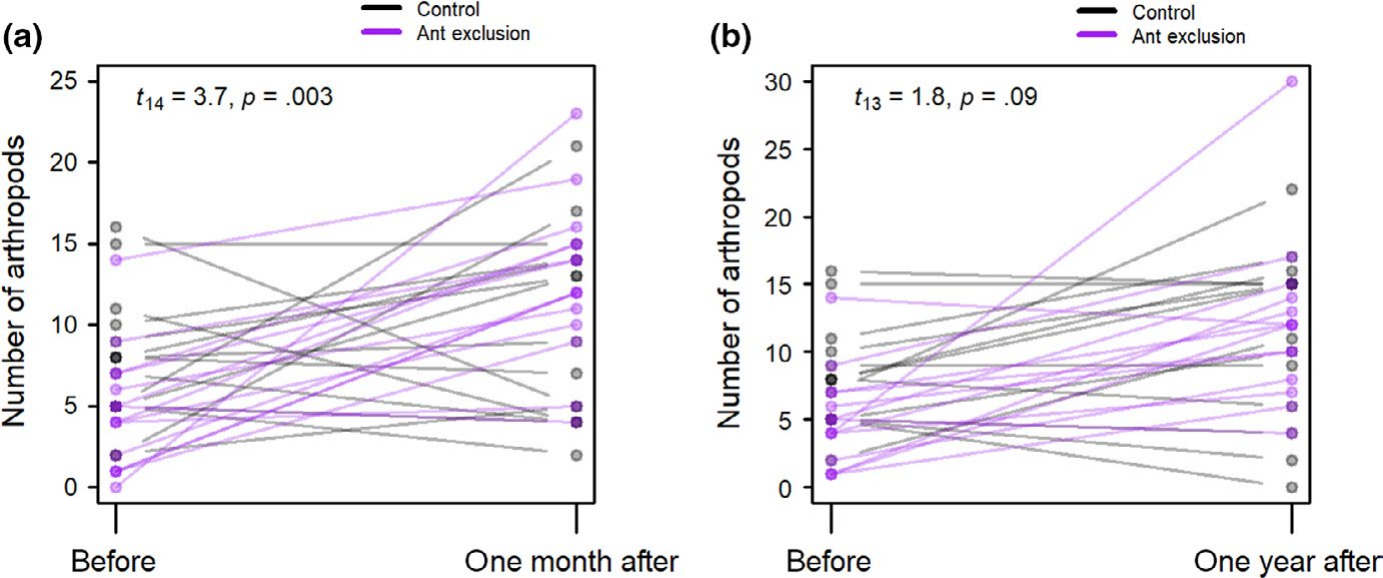
Data from trees paired for size and species, with one member of each pair subject to weaver ant removal and exclusion. Gray lines and dots show control trees and purple lines and dots show treatment trees. Darker dots and lines indicate multiple overlapping points. (a) Number of arthropods (excluding ants and suborder Homoptera) before and 1 month after weaver ant removal and exclusion experiment (*N* = 30 trees). (b) Number of arthropods (excluding ants and suborder Homoptera) before and 1 year after weaver ant removal and exclusion experiment (*N* = 28 trees)

One month after weaver ant removals and exclusion, the numbers of arthropods (excluding ants and homopterans) had increased from 4.67 ± 0.95 to 12.73 ± 1.26 *SE*, *N* = 15, while controls showed no significant change (before: 7.73 ± 1.06 *SE*, after 1 month: 9.8 ± 1.51 *SE*, *N* = 15). Overall, the difference between the change over time in treatment and control trees was significant (paired *t* test, *N* = 15, *p* = .018; Figure 4a). In the following year, the increase in number of arthropods on treatment trees was greater than control trees, but the difference was not statistically significant (Figure 4b; Figure S6). Some of the experimental trees had been recolonized by ants over the course of the year, which might contribute to the reduced effect.

**FIGURE 5:**
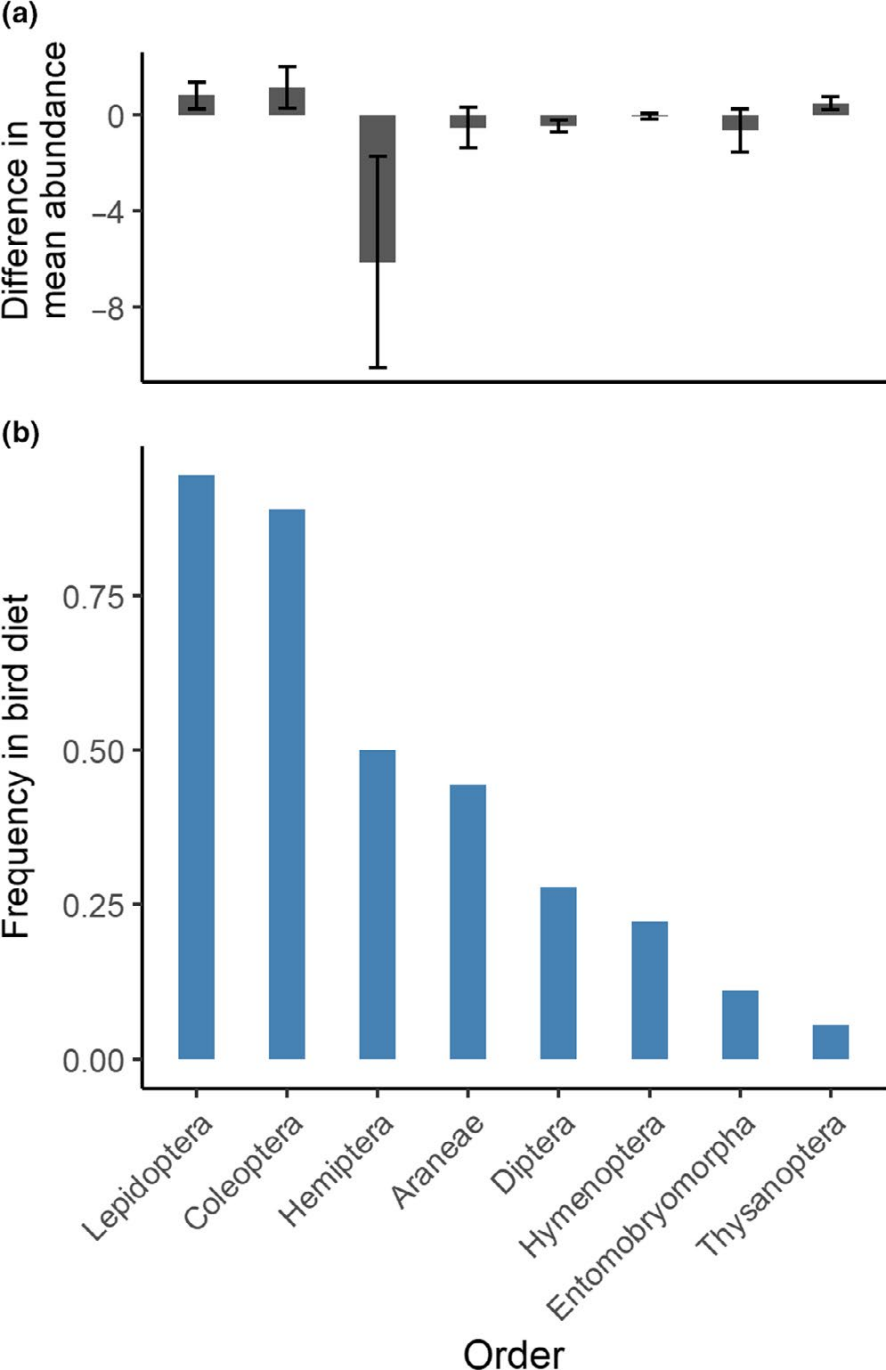
(a) Difference in mean abundance of major arthropod orders in control and treatment trees 1 month after ant exclusion and removal (derived from the data in Figure S7, the error bars indicate standard errors). (b) Frequency of different arthropod orders in bird diets at low elevations (proportion of individual birds with at least one sequence from that order, *N* = 18 individuals)

In the experimental treatments, two taxonomic orders of insects were responsible for the increase on experimental trees with respect to controls (Figure 5a). Lepidoptera increased significantly in abundance (Lepidoptera: treatment 1.0 ± 0.5 *SE*, control −0.5 ± 0.3 *SE*, *N* = 15, paired *t* test *p* = .01), and Coleoptera showed a large increase that was close to significance (treatment 1.3 ± 0.9 SE, control −0.3 ± 0.4 *SE*, *p* = .13). These orders are also the most frequent components of bird diets at all elevations (Figure 5b; Figure S3). On the other hand, Hemiptera decreased after weaver ant exclusion (paired *t* test *N* = 15; treatment −8.8 ± 5.5 *SE*, control 4.3 ± 4.9 *SE*, *p* = .09). This decrease is expected given that it contains the suborder Homoptera and weaver ants form mutualistic associations with Homopterans (Crozier et al., 2009; Peng & Christian, 2005).

## 4 DISCUSSION

In this study, we set out to ask if weaver ants were likely to compete with birds for arthropods at low elevations in eastern Himalaya. Our results show significant diet overlap between weaver ants and birds. Moreover, we found lower arthropod abundance on trees with weaver ants and confirmed experimentally that weaver ant removal led to increased abundance of arthropods. This is important because a greater abundance of arthropods at mid‐elevations correlates with the presence of many small insectivorous bird species at these elevations (Price et al., 2014). Further, the two most common arthropod orders in bird diets at low elevations were the ones to increase most in abundance after weaver ant removal. Since weaver ants are found only at low elevations, they could contribute to the presence of fewer arthropods at these elevations rather than higher up. Together, these results suggest that weaver ants reduce food availability for birds at low elevations in the eastern Himalaya.

Our molecular diet analyses showed overlap in the diet of weaver ants and low elevation birds at all taxonomic levels and overlap in the diet of weaver ants and mid‐elevation birds at higher taxonomic levels. While many previous studies have presented evidence for competition between birds and ants (Table S1), this appears to be the first study that has quantified overlap in diet between an ant species and insectivorous songbirds. Our work also demonstrates the utility of molecular diet analyses to examine dietary niche overlap between distantly related organisms. Studies of dietary niche partitioning among species are increasingly using molecular tools to get fine‐scale taxonomic information on diet composition (Arrizabalaga‐Escudero et al., 2018; Brown et al., 2014; Kartzinel et al., 2015; Razgour et al., 2011). Due to PCR and sequencing biases, these methods may not give accurate information on the quantity of dietary items (Pompanon et al., 2012; Shokralla, Spall, Gibson, & Hajibabaei, 2012). Still, we think that information on the identity and frequency of dietary items obtained using these methods can be very useful to understand the diversity patterns of dietary guilds along environmental gradients.

Reduced herbivory and reduced abundance of large arthropods on trees with weaver ants have been demonstrated previously. Asian weaver ants have been long used as a biological control agent and are still used to control pest populations in orchards (Peng & Christian, 2005; Thurman, Northfield, & Snyder, 2019; Van Mele, 2008; Way & Khoo, 1992). A recent review found that weaver ants significantly reduce pest populations on tropical tree crops (Thurman et al., 2019). Offenberg, Nielsen, MacIntosh, Havanon, and Aksornkoae (2004) showed that a leaf beetle species avoided eating leaves with weaver ant pheromones on them. In mangrove forests, the presence of weaver ants is negatively correlated with leaf damage due to herbivores (Offenberg, Havanon, Aksornkoae, Macintosh, & Nielsen, 2004), as we also found in this study. More broadly, many plant species form strong mutualistic associations with ants to reduce the risk of herbivory by offering them rewards such as food bodies, extra‐floral nectaries, and domatia (i.e., nesting sites; Chomicki, Staedler, Schönenberger, & Renner, 2016; Fiala, Maschwitz, Pong, & Helbig, 1989; Janzen, 1966; Webber, Moog, Curtis, & Woodrow, 2007). Even facultative or opportunistic ant–plant interactions are known to reduce herbivory and deter other arthropods from plants occupied or visited frequently by ants (Bentley, 1977; Chamberlain & Holland, 2009; Fiala, Grunsky, Maschwitz, & Linsenmair, 1994; Rosumek et al., 2009; Trager et al., 2010).

We suggest that the relatively high arthropod abundance at mid‐elevations in the eastern Himalaya is partly a consequence of reduced ant predation, but this does not exclude contributions from other factors, including differences in primary productivity (Acharya, Sanders, Vijayan, & Chettri, 2011), plant diversity and density (Acharya et al., 2011) and higher seasonality (Supriya, Moreau, Sam, & Price, 2019). Overall, our results lend support the idea that competition from ants could contribute to mid‐elevational peak in songbird diversity in the eastern Himalaya. A similar link between competition with ants and diversity patterns was recorded by Brown and Davidson (1977) who found complementary diversity patterns in response to annual precipitation in seed‐eating ants and rodents along a north–south gradient in the US. Likewise, Heaney (2001) suggested competition with ants may be responsible for the peak in small mammal diversity in cloud forests in the Philippines. Other studies have shown patterns of complementary diversity patterns between ants and other arthropod groups, such as staphylinid beetles, carabid beetles, and spiders (Halaj, Ross, & Moldenke, 1997; Hölldobler & Wilson, 1990; Noreika & Kotze, 2012). More generally, the near‐absence of ants in cloud forests (Longino et al., 2014) could be an important explanatory factor for the high diversity of many other taxa there.

A recent meta‐analysis of ant species diversity patterns along elevational gradients found some support for a model whereby temperature and precipitation interact to affect ant diversity, that is there is a significant relationship between temperature and ant diversity on 83% of wet mountains compared to only 25% of arid mountains (Szewczyk & McCain, 2016). Still, the question of why ants are so rare in montane cloud forests remains largely unanswered. Previous studies have suggested that the combination of year‐round cool temperatures and humidity make cloud forests unsuitable for ants (Janzen, 1973; Samson et al., 1997; Wheeler, 1917). However, competition from endotherms such as birds and mammals might also contribute to limiting ant distributions, in much the same way that we postulate ants affect birds in the warm and wet lower elevations. Experimental tests to compare the importance of abiotic versus biotic effects in shaping the pattern of ant abundance along elevational gradients are much needed.

Overall, our observational and experimental data suggest that birds and ants compete for arthropod prey at low elevations in the eastern Himalaya. As diversity patterns of taxa shift due to climate change, it is important to monitor these patterns and compare patterns of such distantly related but potentially competing taxa. We advocate for more studies on ecological interactions between distantly related taxa in shaping diversity patterns, because these interactions could dampen (Suttle, Thomsen, & Power, 2007) or enhance the effect of climate change on species abundance and range distributions, depending on the actors involved (Davis, Jenkinson, Lawton, Shorrocks, & Wood, 1998).

## CONFLICT OF INTERESTS

The authors have no competing interests.

## AUTHORS CONTRIBUTIONS

All authors conceived and designed the study. K.S. carried out fieldwork, molecular laboratory work, data analyses and drafted the manuscript. C.S.M. and T.D.P critically revised the manuscript. All authors gave final approval for publication and agree to be held accountable for the work performed therein.

## ETHICS

This study was approved by Institutional Animal Care and Use Committee at the University of Chicago (ACUP #71393). We also received permission for our fieldwork from the West Bengal Forest Department (1423/WL/4R‐1(Part‐XII)/2014 and 2702/4R‐6/2015).

### Open Research Badges

This article has earned an Open Data Badge for making publicly available the digitally‐shareable data necessary to reproduce the reported results. The data is available at https://doi.org/10.5281/zenodo.2651358

## Supporting information

Supplementary Material

Table S1‐S7

## Data Availability

All data and code used for analyses is available on a GitHub repository (https://doi.org/10.5281/zenodo.2651358).
