# Supplementary material for: Competition with insectivorous ants as a contributor to low songbird diversity at low elevations in the eastern Himalaya
Source: Ecol Evol. 2020 Mar 30;10(10):4280–90. doi: 10.1002/ece3.6196 (PMC7246197; doi:10.1002/ece3.6196)
Supplement: Supplementary file 1 — Supplementary Material [file ECE3-10-4280-s001.docx]

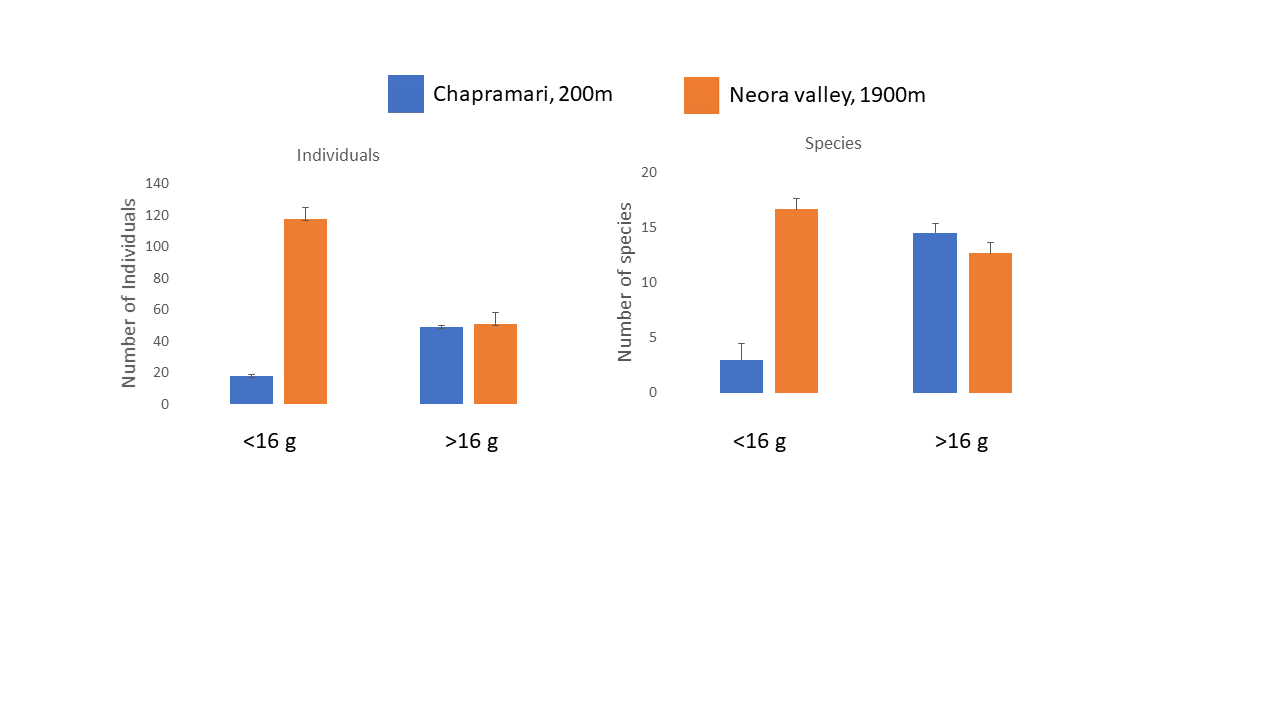


**Figure A1** Number of insectivore individual birds (left) and species (right) on 5ha. grids at the two study sites, divided into small and large categories. Data from Price et al. (2014), based on 2012 censuses. Standard errors are based on replicates across two observers (Chapramari, N = 2), and two adjacent grids, one surveyed by one observer and one by two (Neora Valley, N =3).

**
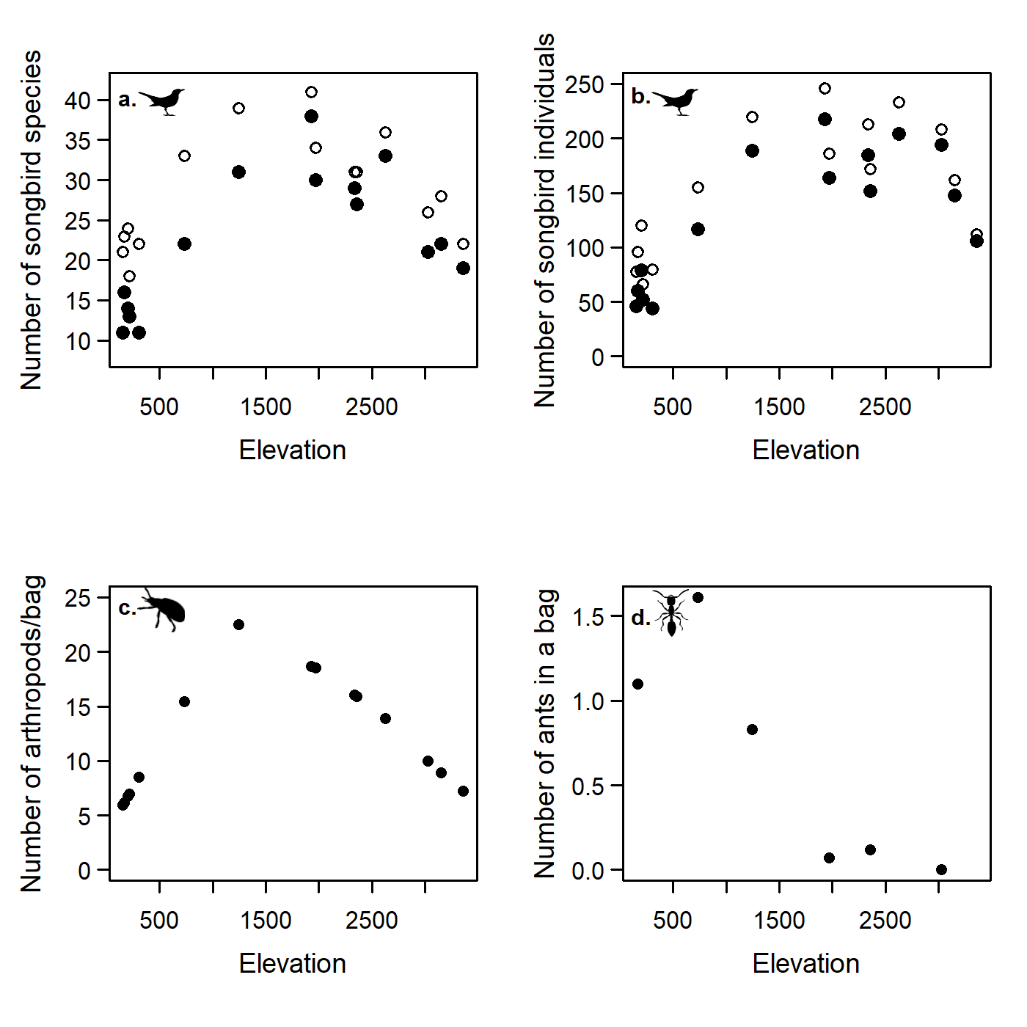
**

**Figure A2** Patterns of abundance and species richness along an elevational gradient in the eastern Himalaya. a. Mid-elevational peak of songbird species richness, as censused in 5ha. grids. b. Mid-elevational peak in number of songbird individuals c. Mid-elevational peak in arthropod abundance d. Decline in ant abundance with elevation (redrawn from data in Price et al [1] and Ghosh-Harihar [2])


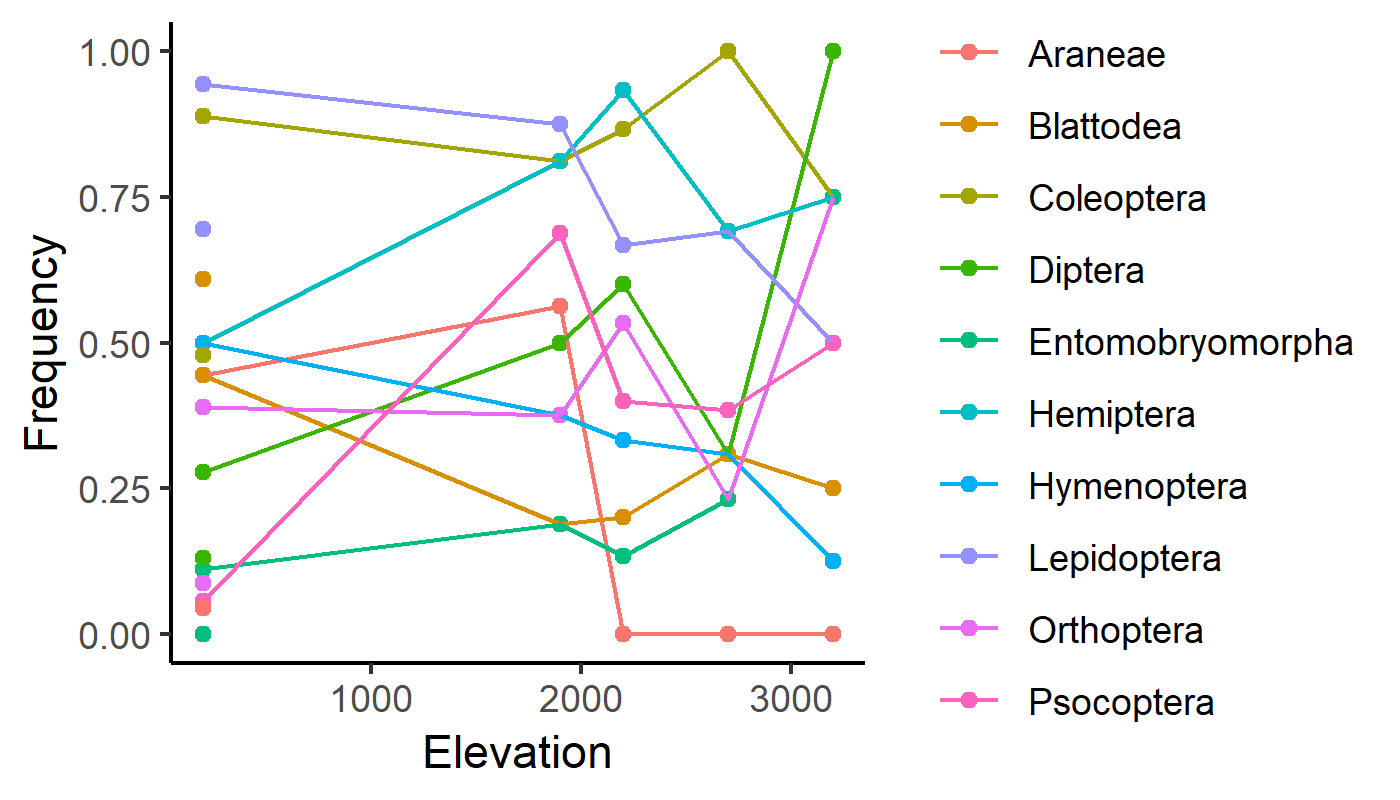


**Figure A3** Patterns of frequency of 10 arthropod orders in bird diet at 200m, 1900m, 2200m, 2700m and 3200m in the eastern Himalaya. Additional unconnected points at 200m elevation show the frequency of the same orders in weaver ant diet.


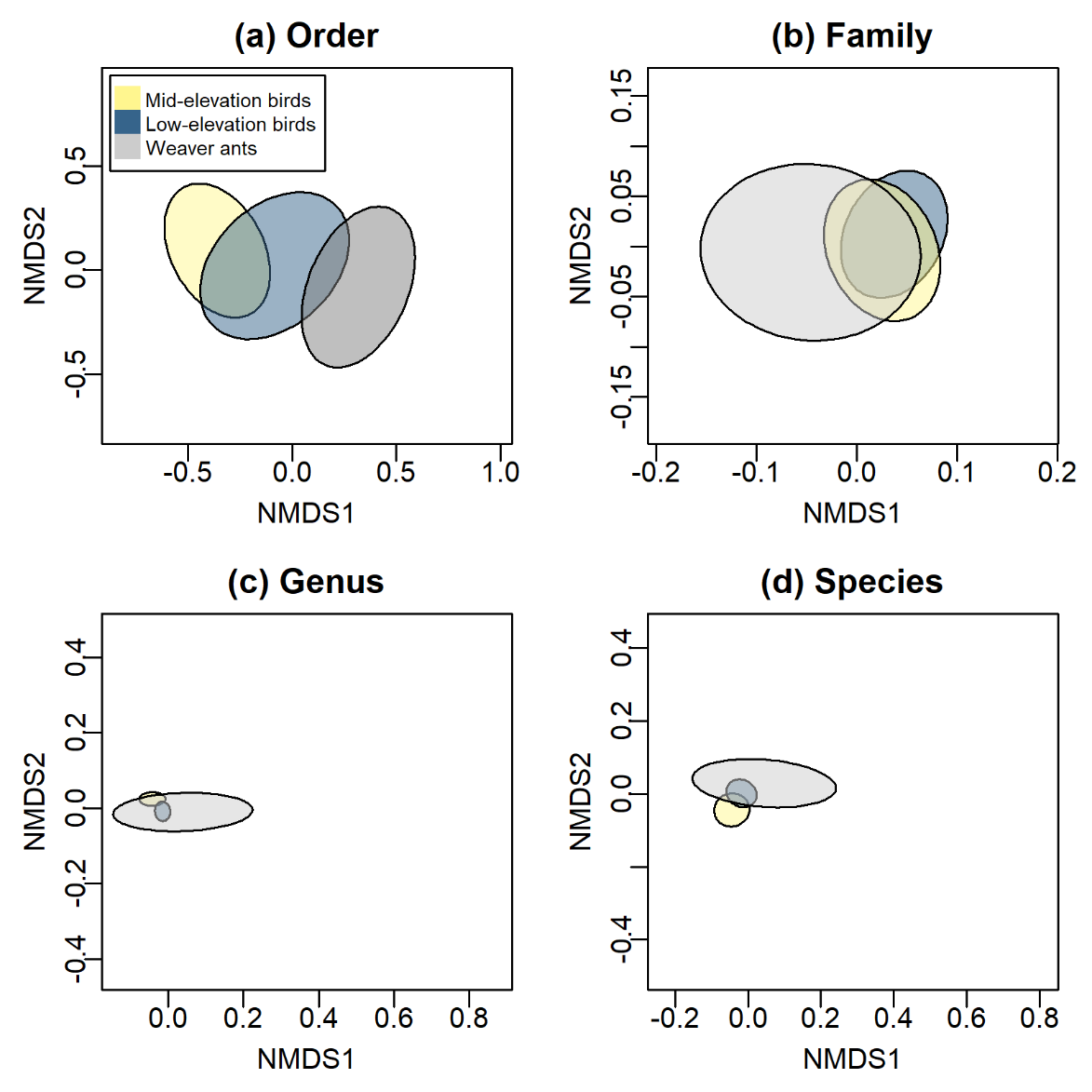


**Figure A4** NMDS plots showing diet overlap between weaver ants (“Oecophylla”), birds at 200m elevation (“Chapramari”) and birds at 2000m elevation (“Bhote”)


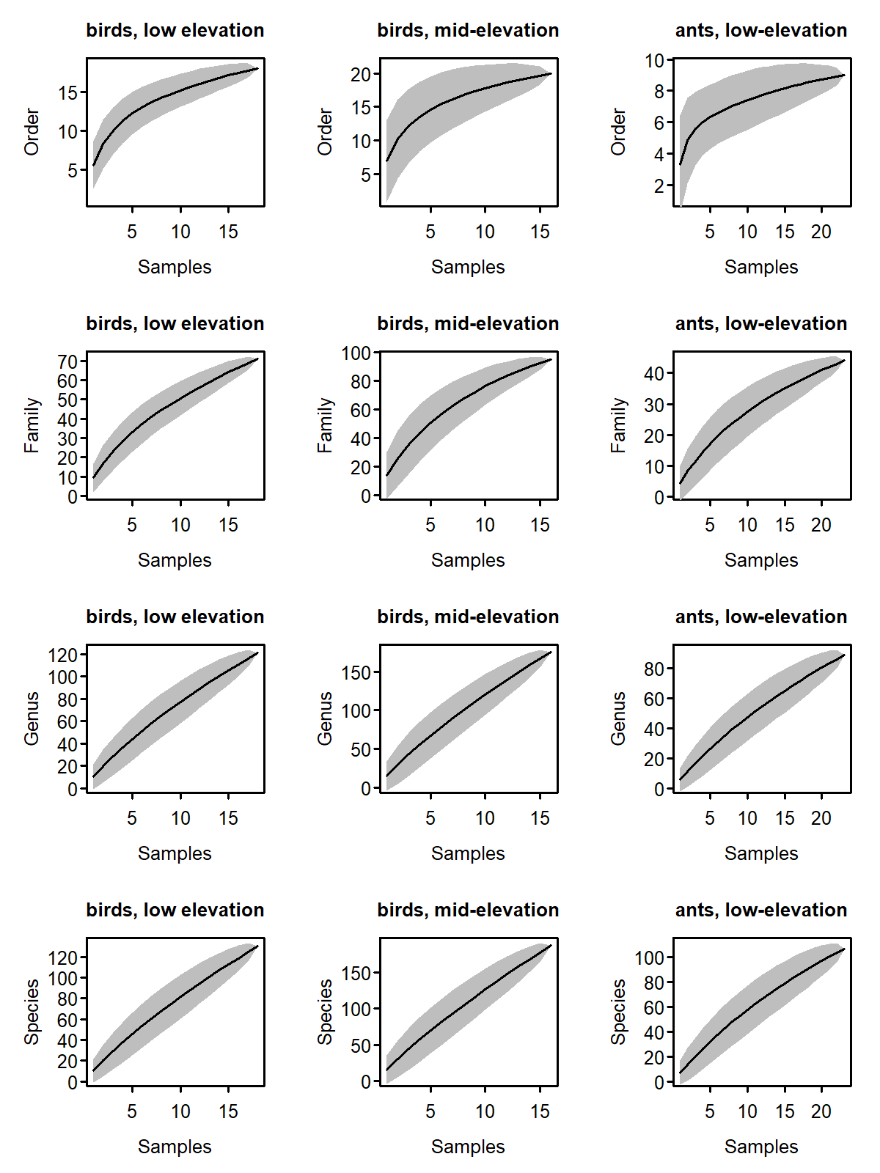


**Figure A5** Taxa accumulation curves at order, family, genus and species levels for the diets of birds at low and mid-elevations and Asian weaver ants.


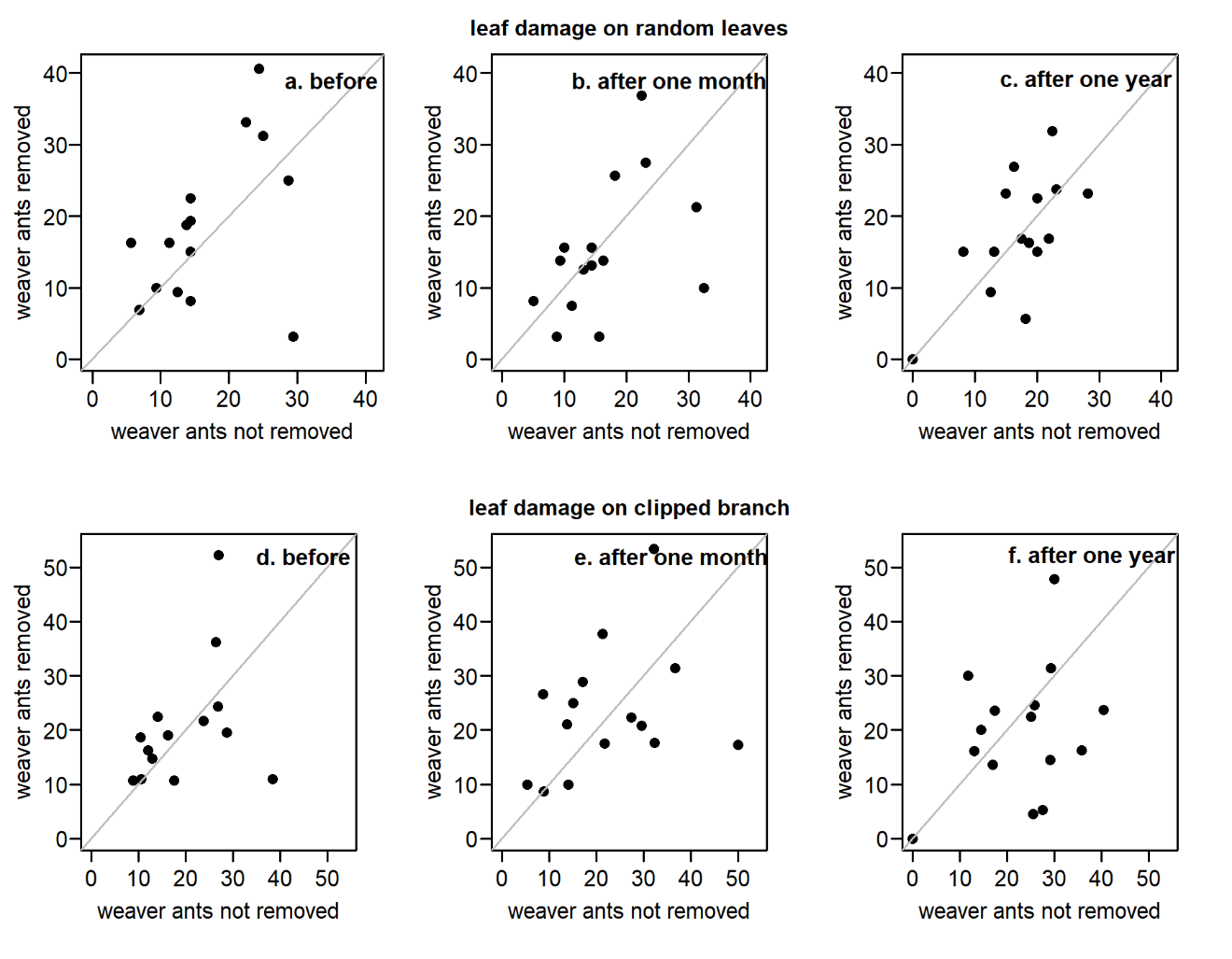


**Figure A6** Leaf damage on each pair of control and treatment trees before (a,d), one month after (b,e) and one year after (c,f) weaver ant removal and exclusion. The grey line shows a slope of 1.0, i.e. points on this line indicate pairs that do not differ in arthropod abundance. (a-c) percent leaf damage estimated using either 16 leaves from different parts of the tree (d-f) percent leaf damage estimated on a clipped branch (all P > 0.05)

**Supplementary methods**

**Effect of weaver ants on arthropod abundance**

We sampled 10 pairs of trees in June 2014 and 7 other pairs of trees in May 2015. To measure arthropod abundance, we beat the foliage of a tree with a stick, and collecting all the insects that fell on an upturned umbrella (≈ 100cm diameter), which is similar to the method used by Piñol et al. [3]. We beat the foliage three times before collecting arthropods in vials containing 95% ethanol and repeated the process at another part of the tree. Later in the field camp, we counted all the collected arthropods, measured body length to the nearest mm, and classified them to taxonomic order. To control for observer bias, the person counting the arthropods was unaware of the presence or absence of weaver ants on the source tree. We used two methods to assess leaf damage: (1) visual estimation of leaf damage (% removed) on 10 leaves from different parts of each tree, and (2) visual estimation of leaf damage on each leaf on a short (~0.5m) clipped branch of the tree. Leaf damage may not accurately represent herbivory when leaf lifespans are different among tree species and habitats [4] and may not reflect arthropod abundance if the arthropod communities on the trees are very different and have different herbivory rates. However, using similar-sized trees belonging to the same species in similar habitats for comparison should control for these confounding factors.

For the weaver ant removal and exclusion experiment, we first located pairs of trees of the same species with roughly the same height and girth that had weaver ants and were not connected to other trees at the canopy level. In some cases, we had to prune the branches of a tree slightly to isolate it from other trees in the area. We randomly assigned trees in each pair to experimental or control treatment using coin tosses. We measured arthropod abundance at each of these trees using beating (as described in the previous paragraph) and branch-clipping [5]. In the branch clipping method, we used a tree pruner to clip a short branch from a tree and let it fall on an upturned umbrella and collected all the arthropods from the surface of the umbrella and from the clipped leaves. We applied treatments to 15 pairs of trees in May 2015 and then measured arthropod abundance and leaf damage at each of the trees in June 2015 using the same methods to assess the effect of weaver ant removal and exclusion one month after the treatment. We checked the trees every week during the field season in April-June 2015 to ensure the treatments were working and re-applied Tanglefoot or added cotton to the crevices as and when necessary. Between July 2015- April 2016, our assistants checked the trees monthly to maintain the treatments. However, we were unable to maintain the treatments as weaver ants reappeared on four of the 14 treatment trees and disappeared from seven of the 14 control trees (one of our initial pairs was destroyed by an elephant).

**Molecular diet analysis**

We used mist nets to catch birds in May & June 2015 and 2016. Upon extracting birds from the net, we put them in a brown bag for one minute, weighed them, took them out, measured their wing and tarsus length and released them. After the bird was released, we tore up the brown bag, scraped the faeces off and put them in a 2 ml plastic tube with 95% ethanol. Average processing time for a bird was about two minutes. To examine weaver ant diet, we collected food from 25 colonies either for an hour or until we had collected 10 items, whichever happened first. We extracted DNA from these samples by taking a small part of each of the prey items, crushing it and then using the Qiagen DNeasy blood and tissue kit and following the manufacturer’s protocol for extraction. For our PCR reactions, we used 10μL of the DNA extract from bird feces or 2μL of the DNA extract from weaver ant food samples as the template (see Supplementary methods for detailed protocol). We visualized 3μL of the PCR products on a gel to identify successful amplifications.

We followed the dada2 pipeline [6] to filter and trim our sequences, identify unique sequences, merge paired reads, remove chimeras and construct an amplicon sequence variant (ASV) table. Because our amplicons varied in length, we did not trim sequences based on the quality profiles and instead just trimmed 20 bp from both the forward and reverse sequences to remove the primer sequences. Using the ASV table generated by dada2, we created a FASTA file with all the unique sequences recovered from our samples. We used blastn in blastplus to BLAST this FASTA file against the NCBI nr database with word size (i.e. length of initial region of exact sequence match) set to 9 bp and e-value set to 1^-10^. Next, we used the NCBI-taxcollector script [7] to get detailed taxonomic information for the top hit of our BLAST results. We filtered out sequences that were identified as bacteria or rotifers and removed potential contaminants such as sequences identified as human. Some of the BLAST matches were obviously incorrect, because the taxon identified by BLAST is not known to occur in the region. For example, our results show the order Vetigastropoda in the diet of birds at low elevation, but Vetigastropoda are exclusively marine. Such misclassifications likely arise from the lack of availability of DNA sequences for organisms in our study area. However, we think the taxonomic identifications are still valuable, because the ASVs that are grouped together likely belong to taxonomic groups closely related to the group they are misclassified into.

We used EcoSimR [8] to estimate diet overlap between weaver ants and birds. EcoSim R is used to estimate niche overlap between species and compare it to a null distribution of niche overlap given information on resource utilization (in columns) by each species (rows). The algorithm randomizes resource utilization for each species by reshuffling the row values and generates a null distribution of niche overlap. In our case, we considered birds at low elevations, birds at mid elevations and weaver ants at low elevations as “species” and used the frequency of occurrence of different orders or families in diets as the resources. All the analyses were done in the R programming environment and in the shell using the R packages dada2 [6], DECIPHER [9], VennDiagram [10], ggplot2 [11], vegan [12] and EcoSimR [8]. All scripts and details of the steps are available on a github repository [13].

**DNA extraction protocol for bird feces stored in ethanol.**

Methodology uses the Qiagen QIAamp DNA Stool Kit, following the “Isolation of DNA from Stool for Pathogen Detection” protocol (June 2012 edition), with some modifications following Zeale *et al*. [14] and additional modifications for our samples.

**1.** Remove ~100-200 mg of faeces (no need to weigh, take typically 2-3 small fragments of faeces) from storage tube and place into a 2 mL round-bottomed centrifuge tube (Eppendorf 2 mL SafeLock tubes are good). Allow ethanol to evaporate off and avoid using faeces that has lots of uric acid on it.

**2.** Add 1.4 mL Buffer ASL to the faecal sample and vortex vigorously for at least one minute.

**3.** Add 20 µL of Proteinase K. Vortex briefly to mix, then heat the suspension for 30 minutes at 70^o^C.

**4.** Vortex for 30 seconds, then centrifuge sample at 13,000 rpm for 1 minute to pellet faecal particles.

**5.** Pipet 1.2 mL of the supernatant into a new 2 mL centrifuge tube. The remaining faecal material can be stored and used for microscopic analysis if required; otherwise discard the pellet.

**6.** Add 1 InhibitEX tablet to the sample and vortex immediately and continuously for 1 minute or until the tablet is completely suspended. Incubate suspension for 1 minute at room temperature to allow inhibitors to absorb to the InhibitEX matrix.

**7.** Centrifuge sample at 13,000 rpm for 3 minutes to pellet inhibitors bound to InhibitEX matrix.

**8.** Pipet all the supernatant (typically 400-600 µL) into a new 1.5 mL centrifuge tube and discard the pellet. Centrifuge the sample at full speed for 3 minutes. Transfer of small quantities of pellet material will not affect the procedure.

**9.** Pipet 20 µL of Proteinase K (either from kit, or a user-supplied 10mg/mL solution) into a new 1.5 mL centrifuge tube.

**10.** Pipet 400 µL of supernatant from step 8 into the 1.5 mL tube containing proteinase K.

**11.** Add 400 µL of Buffer AL and mix well by vortexing for 15 seconds. Don’t add the proteinase K directly to buffer AL.

**12.** Incubate at 70 ^o^ C for 15-30 minutes.

**13.** Add 400 µL of ethanol (96-100%) to the lysate and mix well by vortexing. Centrifuge briefly to remove any liquid from the lid of the tube.

**14.** Carefully apply 600 µL of the lysate to a QIAmp spin column (in a 2 mL collection tube) without moistening the rim. Centrifuge at 13,000 rpm for 1 minute. Place spin column in a new 2 mL collection tube and discard the tube containing the filtrate.

**15.** Repeat step 14 using the remaining liquid from step 13.

**16.** Carefully open the spin column and add 500 µL of Buffer AW1. Centrifuge at 13,000 rpm for 1 minute. Place spin column in a new 2 mL collection tube and discard the tube containing the flow-through.

**17.** Carefully open the spin column and add 500 µL of Buffer AW2. Centrifuge at 13,000 rpm for 2 minutes. Discard tube containing the flow-through.

**18.** Place spin column in a new 2 mL collection tube. Centrifuge at 13,000 rpm for 1 minute. Discard tube containing flow-through.

**19.** Transfer the spin column into a new 1.5 mL centrifuge tube. Using a low-bind tube will minimise DNA loss through absorption to tube walls (Eppendorf DNA LoBind tubes are good). Pipet 100 µL of Buffer EB (not supplied in kit; EB = 10 mM Tris) directly onto the spin column membrane. Incubate for 30 minutes at room temperature, then centrifuge at 13,000 rpm for 1 minute to elute DNA.

**PCR protocol**

Each 25 µL PCR reaction contained the following reagents for DNA extracts from bird faeces:

| **Reagent** | **µL Per sample** |
| --- | --- |
| PCR water | 6.4 |
| buffer (10x) | 2.5 |
| dNTPs (8 mM) | 2.5 |
| lns16S_1-F (10 µM) | 1.2 |
| lns16S_1-R (10 µM) | 1.2 |
| BSA | 1 |
| Roche taq (5U/µL) | 0.2 |
| DNA | 10 |

For DNA extracts from ant prey items, we reduced the DNA template to 2 µL and increased PCR water to 14.4 µL.

Thermal cycler conditions:

| Initial Denature: 95°C for 10 min |
| --- |
| Denature: 95°C for 30s |
| Anneal: 50°C for 30 s |
| Extension: 72°C for 30s |
| Final Extension: 72°C for 10 min |
| Number of cycles: 40 |

**Literature cited**

1. Price TD *et al.* 2014 Niche filling slows the diversification of Himalayan songbirds. *Nature* **509**, 222–225. (doi:10.1038/nature13272)

2. Ghosh-Harihar M. 2013 Distribution and abundance of foliage-arthropods across elevational gradients in the east and west Himalayas. *Ecol. Res.* **28**, 125–130. (doi:10.1007/s11284-012-1000-2)

3. Piñol J, Espadaler X, Cañellas N. 2012 Eight years of ant-exclusion from citrus canopies: effects on the arthropod assemblage and on fruit yield. *Agric. For. Entomol.* **14**, 49–57. (doi:10.1111/j.1461-9563.2011.00542.x)

4. Moles AT *et al.* 2011 Putting plant resistance traits on the map: a test of the idea that plants are better defended at lower latitudes. *New Phytol.* **191**, 777–788. (doi:10.1111/j.1469-8137.2011.03732.x)

5. Ozanne C. 2005 *Insect sampling in forest ecosystems*. John Wiley & Sons.

6. Callahan BJ, McMurdie PJ, Rosen MJ, Han AW, Johnson AJA, Holmes SP. 2016 DADA2: High resolution sample inference from Illumina amplicon data. *Nat. Methods* **13**, 581–583. (doi:10.1038/nmeth.3869)

7. Dias R, Xavier MG, Rossi FD, Neves MV, Lange T a. P, Giongo A, De Rose C a. F, Triplett EW. 2014 MPI-blastn and NCBI-TaxCollector: Improving metagenomic analysis with high performance classification and wide taxonomic attachment. *J. Bioinform. Comput. Biol.* **12**, 1450013. (doi:10.1142/S0219720014500139)

8. Gotelli NJ, Hart EM, Ellison AM. 2015 *EcoSimR: Null model analysis for ecological data. R package version 0.1.0.* See http://github.com/gotellilab/EcoSimR doi:10.5281/zenodo.16522.

9. Wright E. 2016 Using DECIPHER v2.0 to Analyze Big Biological Sequence Data in R. *R J.* **8**, 352–359.

10. Chen H, Boutros PC. 2011 VennDiagram: a package for the generation of highly-customizable Venn and Euler diagrams in R. *BMC Bioinformatics* **12**, 35. (doi:10.1186/1471-2105-12-35)

11. Wickham H. 2011 ggplot2. *Wiley Interdiscip. Rev. Comput. Stat.* **3**, 180–185. (doi:10.1002/wics.147)

12. Oksanen J *et al.* 2018 *vegan: Community Ecology Package.* See https://CRAN.R-project.org/package=vegan.

13. Supriya K, Price TD, Moreau CS. 2019 Weaverantsbirds: Data & scripts for ProcB submission.

14. Zeale MRK, Butlin RK, Barker GLA, Lees DC, Jones G. 2011 Taxon-specific PCR for DNA barcoding arthropod prey in bat faeces. *Mol. Ecol. Resour.* **11**, 236–244. (doi:10.1111/j.1755-0998.2010.02920.x)
